# Contralateral Metabolic Activation Related to Plastic Changes in the Spinal Cord after Peripheral Nerve Injury in Rats

**DOI:** 10.1155/2015/438319

**Published:** 2015-09-28

**Authors:** Ran Won, Bae Hwan Lee

**Affiliations:** ^1^Department of Biomedical Laboratory Science, Division of Health Sciences, Dongseo University, Busan 617-716, Republic of Korea; ^2^Department of Physiology, Brain Research Institute, Epilepsy Research Institute, Brain Korea 21 PLUS Project for Medical Science, Yonsei University College of Medicine, Seoul 120-752, Republic of Korea

## Abstract

We have previously reported the crossed-withdrawal reflex in which the rats with nerve injury developed behavioral pain responses of the injured paw to stimuli applied to the contralateral uninjured paw. This reflex indicates that contralateral plastic changes may occur in the spinal cord after unilateral nerve injury. The present study was performed to elucidate the mechanisms and morphological correlates underlying the crossed-withdrawal reflex by using quantitative ^14^C-2-deoxyglucose (2-DG) autoradiography which can examine metabolic activities and spatial patterns simultaneously. Under pentobarbital anesthesia, rats were subjected to unilateral nerve injury. Mechanical allodynia was tested for two weeks after nerve injury. After nerve injury, neuropathic pain behaviors developed progressively. The crossed-withdrawal reflex was observed at two weeks postoperatively. Contralateral enhancement of 2-DG uptake in the ventral horn of the spinal cord to electrical stimulation of the uninjured paw was observed. These results suggest that the facilitation of information processing from the uninjured side to the injured side may contribute to the crossed-withdrawal reflex by plastic changes in the spinal cord of nerve-injured rats.

## 1. Introduction

It has been shown that peripheral nerve injury can cause severe chronic pain in humans [1, 2]. Humans frequently experience neuropathic pain symptoms such as spontaneous burning painful sensations, hyperalgesia (elevated sensitivity to noxious stimulation), and allodynia (painful experience to innocuous stimulation) after peripheral nerve injury.

Occasionally, neuropathic pain symptoms can be observed on the opposite side of the nerve injury in humans or in experimental animal models of neuropathic pain [1–4]. It indicates that the intact side of the body can be sensitive and may produce secondary pain after unilateral nerve injury [5, 6]. This phenomenon has been known as the “mirror image pain.” Although the mechanism of mirror image pain remains unclear, the investigation for possible mechanisms for mirror image pain is an ongoing project. In mirror image pain, IL-6 protein and mRNA in both lumbar and cervical dorsal root ganglia were elevated bilaterally following unilateral chronic compression injury of the sciatic nerve [7]. Also, bilateral changes of cannabinoid receptor type 2 protein and mRNA in the dorsal root ganglia of a rat neuropathic pain model were reported [8].

In our previous study [9], we observed that the rats with unilateral nerve injury showed withdrawal responses of the injured paw to stimuli applied to the contralateral uninjured paw. This phenomenon is fundamentally different from mirror image pain. Therefore, this phenomenon is called “the crossed-withdrawal reflex.” The mechanisms underlying the crossed-withdrawal reflex are still uncertain. For the crossed-withdrawal reflex to appear, the dorsal horn neurons on the intact side need to be activated by applied stimulation and then the ventral horn neurons on the injured side need to be activated by inputs from the intact side. However, the connections between the dorsal horn neurons on the intact side and the ventral horn neurons on injured side are unclear. For example, the information may be transmitted directly from the dorsal horn on the intact side to the ventral horn of the injured side. Otherwise, the input information on the intact side may go indirectly to the ventral horn on the injured side via a relaying part of the spinal cord. Despite the lack of information of a detailed mechanism, the crossed-withdrawal reflex is a very interesting phenomenon. It appears to reflect the central neuroplastic changes, which involve various morphological, electrophysiological, and biochemical processes.

The fact that increased neuronal excitability is linked with increased metabolism has been confirmed by ^14^C-2-deoxyglucose (2-DG) autoradiography, which is a functional mapping technique established by Sokoloff et al. [10]. 2-DG is phosphorylated by hexokinase to form 2-DG-6-phosphate, which is not metabolized further and trapped within activated cells. 2-DG technique allows simultaneous examination of metabolic activity and spatial pattern affected by a stimulus or behavioral condition [11–14]. Recently, Dedeurwaerdere et al. [15] reported patterns of brain glucose metabolism to investigate a clinically functional biomarker by using 2-DG autoradiography technique.

The present study was conducted to elucidate the mechanism of “the crossed-withdrawal reflex” and to clarify the alteration of metabolic activity of this phenomenon using the quantitative 2-DG autoradiography technique.

## 2. Materials and Methods

### 2.1. Neuropathic Model

Male Sprague-Dawley rats weighing between 200 and 250 g were used. All animal experiments were approved by the Institutional Animal Care and Use Committee of Yonsei University Health System. Twelve rats were anesthetized with pentobarbital (Entobar, 50 mg/kg, i.p.). To make a neuropathic injury model, the tibial and sural nerves were cut with fine scissors after a tight ligation with a 6-0 silk thread, while the common peroneal nerve was left intact [16].

### 2.2. Behavioral Tests

Mechanical allodynia was carried out in all of the rats preoperatively and again for two weeks postoperatively. An innocuous mechanical stimulus was applied with a von Frey filament (8 mN of bending force) to the sensitive area of the hind paw. Rats were placed on a metal mesh floor under a transparent plastic dome (8 × 8 × 18 cm). By poking various areas of the paw with a von Frey filament the most sensitive area was determined in advance. Afterwards, the test was conducted 10 times to each hind paw by gently poking the most sensitive spot with the filament. The frequency of foot withdrawal expressed as a percentage was used as the index of mechanical allodynia. Assessments were made on both the injured side and uninjured contralateral side.

To examine the crossed-withdrawal reflex, animals expressing response rates equal or more than 40% in mechanical allodynia were stimulated to the contralateral uninjured paw. To compare to the real stimulation, sham stimulation was applied according to the same procedure as von Frey filament except for actual contact and then the frequency of the paw withdrawal was expressed as shown above. The responses to the actual and sham applications of von Frey filament were compared using the paired* t*-test.

### 2.3. Autoradiography

Five nerve-injured rats and four normal controls weighing 250–350 g were used to conduct 2-DG technique. Under pentobarbital anesthesia (50 mg/kg), thoracic jugular vein catheter (Braintree Scientific, Inc. MA. USA) was inserted into the external jugular vein contralateral to the side of nerve injury. The free end of the catheter was expelled through a subcutaneous tunnel to a small incision in the top of the head. A bolus of ^14^C-2-DG (25uCi) was then injected over 15 sec into the jugular vein 5 min before the uninjured paw was electrically stimulated. Electrical pulses (rectangular pulse, 3–6 mA, 0.4 msec, 0.5 Hz) were delivered for 20 min to bipolar needle electrodes inserted subcutaneously into the receptive field (defined in the behavior test) of the uninjured hind paw. 25 min after the electrical stimulation, the animals were sacrificed by an overdose of pentobarbital and perfused with saline and 10% formalin solution. The spinal cords (L1-L6) were rapidly removed and immediately frozen. Lumbar segments of the spinal cord were sectioned into 20 *μ*m slices using a cryostat (Leica, Wetzlar, Germany) at −20°C.

The sections were attached to a cardboard and were exposed with precalibrated [^14^C]-methyl methacrylate standards (Amersham, Piscataway, NJ, USA) to X-ray film (Kodak BioMax MR) for 10 days. For quantitative analysis, the spinal cord gray matter was divided into 4 regions: the dorsal horn (corresponding approximately to laminae I-IV), the intermediate horn (laminae V-VI), the ventral horn (laminae VII-IX), and the central gray (lamina X). The selected regions were digitized using computer-generated templates for each spinal lumbar segment from L1 to L6. The digitized images were quantified using the MetaMorph Imaging System (Universal Image Co., Downingtown, PA, USA).

### 2.4. Statistical Analysis

Data were expressed as means ± S.E.M. Differences in changes of neuropathic pain behaviors following nerve injury were tested using Friedman's two-way ANOVA by ranks, followed by post-hoc pairwise comparisons.

The frequency of the paw withdrawal responses to the actual applications of von Frey filament and sham stimulation was compared using the paired* t*-test. The crossed-withdrawal reflex developing after nerve injury was analyzed using Friedman's two-way ANOVA by ranks followed by post hoc pairwise comparisons. Pearson's product-moment correlation coefficient between responsiveness of the injured and uninjured paws to von Frey filament of the uninjured side was analyzed using* t*-test.

To evaluate the lateralization of information processing following electrical stimulation of the uninjured side of the spinal cord, data from the ventral horns both ipsilateral and contralateral to electrical stimulation were analyzed using the cross ratio, which is as follows: [metabolic activity in the contralateral ventral horn]/[metabolic activity in the ipsilateral ventral horn] × 100.

Data were expressed as means ± S.E.M. at each level of the lumbar spinal cord. Cross ratios of the nerve-injured rats and those of normal control rats were compared at each spinal level using the independent* t*-test.

## 3. Results 

### 3.1. Development of Mechanical Hypersensitivity

**Figure 1:**
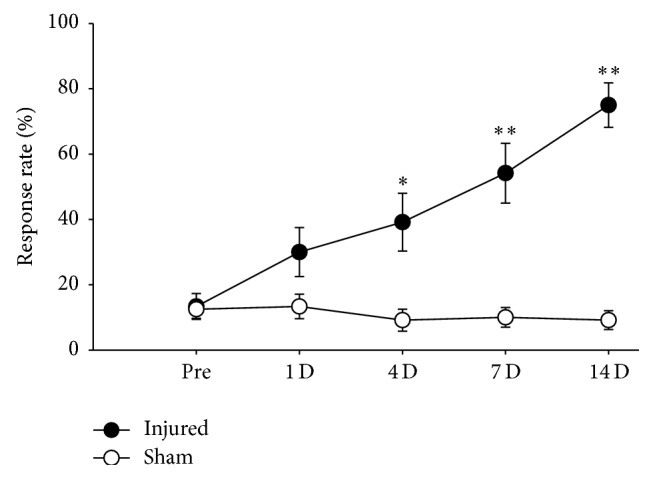
Development of mechanical allodynia in rats with injury to the tibial and sural nerves. Response rate to a von Frey filament was used as an index of mechanical allodynia. Data were expressed as means ± S.E.M. Abscissa was marked as Pre for preoperative time and D for postoperative days (Sham: sham-operated control rats, Injured: neuropathic rats). Asterisks (*∗*) indicate significant differences compared to preoperative values, according to Friedman's repeated ANOVA on ranks followed by Dunnett's multiple comparison test (^*∗*^ *P* < 0.05, ^*∗∗*^ *P* < 0.01).

The result of mechanical allodynia is shown in Figure 1. The response rate to a von Frey filament was used as an index of mechanical allodynia. As shown in Figure 1, the rats were rarely responsive to a von Frey filament prior to the surgery. The responsiveness of the injured foot gradually increased from the first day and reached its highest level on the 14th day postoperatively.

### 3.2. Behavioral Crossed-Withdrawal Reflex

**Figure 2:**
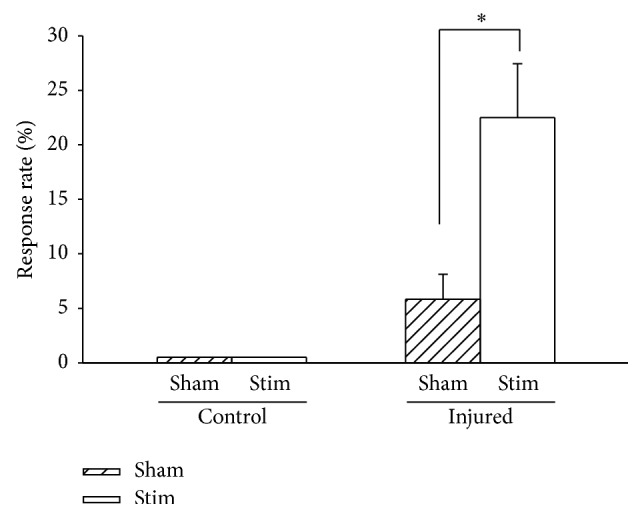
Crossed-withdrawal reflex measured by the response of the injured paw to stimulation of the uninjured paw on day 14 postoperatively. The frequency of the paw withdrawal was expressed as means ± S.E.M. and compared between actual applications (Stim) of von Frey filament and sham stimulation (Sham). (Control: control rats, Injured: neuropathic rats). An asterisk (*∗*) indicates significant differences between actual applications of stimuli and sham stimulation, according to the paired* t*-test (^*∗*^ *P* < 0.05).

In Figure 2, the result of the crossed-withdrawal reflex is shown by expressed responsiveness of the injured paw to the application of a von Frey filament to the uninjured side. The frequency of the paw withdrawal was compared between the actual stimulation of von Frey filament and sham application. The crossed-withdrawal reflexes were apparently higher than the responsiveness of the injured paws to the sham stimulation (*P* < 0.05, paired* t*-test) but the reflex was not observed in normal control animals.

### 3.3. Effects of Nerve Injury on Metabolic Activities in the Spinal Cord

**Figure 3:**
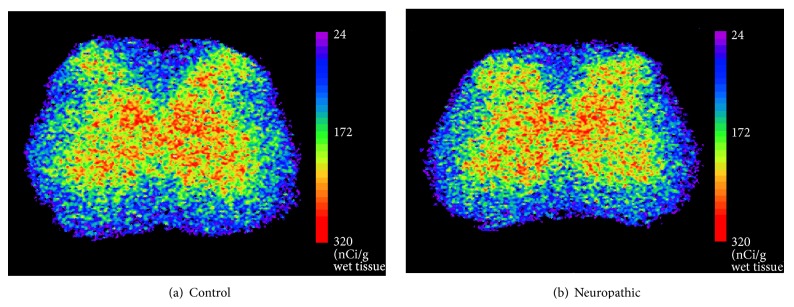
Representative pseudocolor-enhanced images showing metabolic activities in the L3 lumbar spinal cord of control (a) and neuropathic (b) rats after peripheral stimulation. The right side of the spinal cord is ipsilateral to the electrical stimulation of the hind paw. Note the increased 2-DG uptake in the contralateral ventral horn of the neuropathic rat compared to the ipsilateral ventral horn. On the contrary, 2-DG uptake increased in the ipsilateral ventral horn of the control rat compared to the contralateral ventral horn.

To observe any increased crossing input from the uninjured side to the injured side in the spinal cord, the metabolic activities of the lumbar spinal cord in normal controls and nerve-injured rats that expressed cross-withdrawal reflexes were compared. Electrical stimulation of the uninjured paw of neuropathic rats produced significantly increased 2-DG uptake in contralateral ventral horn in the lumbar spinal cord; but, in normal controls, upregulation of 2-DG uptake was produced in the ipsilateral ventral horn where the electrical stimulation was applied (Figure 3).

**Figure 4:**
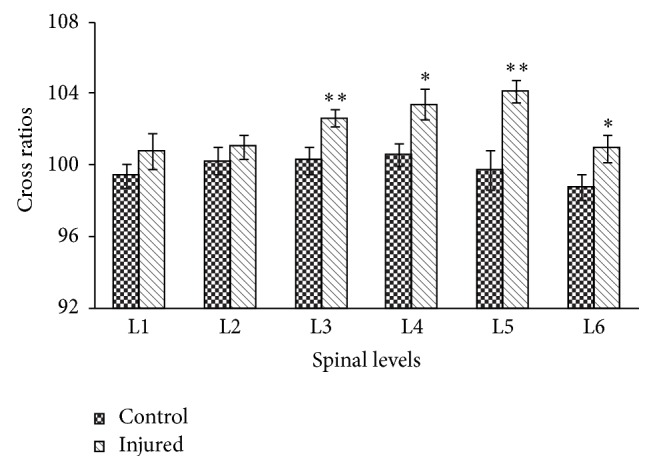
Comparison between cross ratios at different lumbar levels of the spinal cord in normal and neuropathic rats following electrical stimulation of the paw. Cross ratios were calculated as follows: [metabolic activity in the contralateral ventral horn]/[metabolic activity in the ipsilateral ventral horn] × 100 (Control: control rats, Injured: neuropathic rats). Asterisks (*∗*) indicate significant differences between cross ratios at different lumbar levels of the spinal cord in normal and neuropathic rats, according to the independent* t*-test (^*∗*^ *P* < 0.05, ^*∗∗*^ *P* < 0.01).

Cross ratios from different levels of the spinal cord are compared in Figure 4. Of the lumbar segments, L3 to L6 segments in nerve-injured rats were associated with significantly higher cross ratios than normal controls (*P* < 0.05, independent* t*-test). In the L1-L2 segments, the cross ratios of nerve-injured rats tended to be slightly higher but were not significantly different from controls (*P* > 0.05, independent* t*-test).

## 4. Discussion

After nerve injury, bilateral allodynia including original pain and mirror image pain can be found in experimental animal models as well as human patients. It has been shown that the mirror image pain develops in a minority of human patients but it recurs frequently [17].

Contralateral changes resulting from ipsilateral pain symptoms have also been reported through different experimental paradigms in the absence of lesions of the nervous systems. Subcutaneous injection of dilute formalin solution into a hind paw induced a mirror-image-like pain, and the rats frequently licked the contralateral untreated hind paw [18]. A similar phenomenon was reported using a unilateral inflammation model in the rat ankle [19]. These observations suggest that initial painful states on one side may produce hypersensitivity on the contralateral uninjured side following unilateral injury. Therefore, the secondary pain on the other side can be seen as a “mirror image pain.” However, the mechanisms underlying mirror image pain may be different from those of original pain ipsilateral to injury as the original ipsilateral pain was relieved by intravenous lidocaine but not the mirror image pain [20].

In the present study, the crossed-withdrawal reflex represents information processing from input on the uninjured side to output on the injured side. This phenomenon was morphologically determined by using the 2-DG autoradiography technique. As determined by cross ratio using the 2-DG technique, the magnitude of the contralateral metabolic 2-DG activity over the ipsilateral activity by the stimulation of the uninjured side in the neuropathic rat was greater than that in the normal control animals. This result indicates that information processing from input on the uninjured side to output on the injured side must be facilitated in rats with nerve injury. This result provides a morphological and metabolic evidence for the behavioral cross-withdrawal reflex.

Similarly, it was found that spinal cord glucose consumption increased both contralaterally and ipsilaterally after chronic constriction injury of the unilateral sciatic nerve [11, 12]. Contralateral increase of 2-DG uptake in the spinal cord was also found after unilateral noxious heat stimulation of a hind limb in cats [21] and after formalin injection into the hind paw of rats [19], but there has been no report that has shown facilitated metabolic activity from input on the uninjured side to output on the injured side. Even though it is not exactly in agreement with the report that spinal cord activity may be altered in chronic pain states [22], our own results support the hypothesis that the observed metabolic pattern in the spinal cord is mainly due to information processing from input on the uninjured side to output on the injured side. The present 2-DG data are in accordance with the behavioral and electrophysiological findings of the crossed-withdrawal reflex [9].

**Figure 5:**
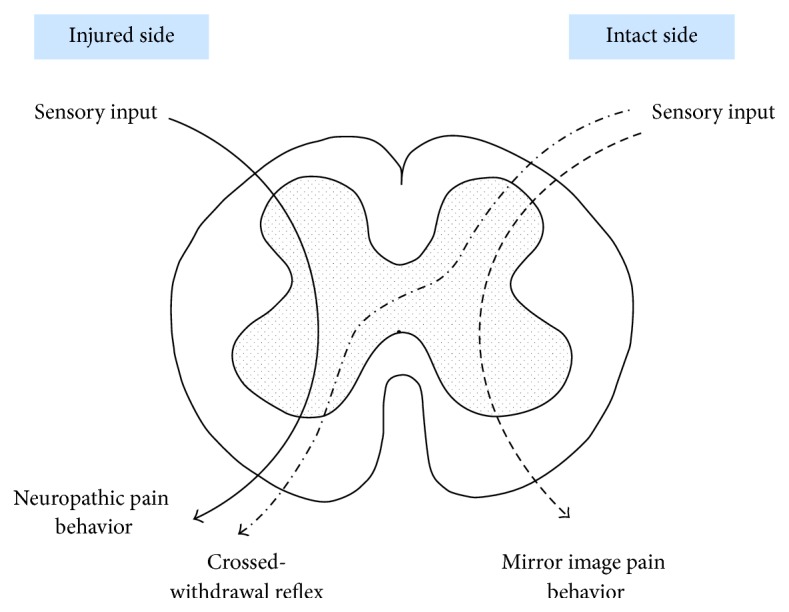
Diagram showing the neuropathic behavior, mirror image pain behavior, and crossed-withdrawal reflex. The crossed-withdrawal reflex is the increased responsiveness of the injured side to stimulation on the uninjured side. In terms of input-output information processing, conventional neuropathic pain and mirror image pain behaviors are represented unilaterally. However, the crossed-withdrawal reflex is represented contralaterally. In other words, input through the dorsal root traverses across the midline of the spinal cord toward the contralateral ventral horn.

Our previous study [9] reported that rats show the crossed-withdrawal reflex after unilateral peripheral nerve injury. The crossed-withdrawal reflex is known as the increased responsiveness on the injured side to stimulation on the uninjured side, indicating neuroplastic changes in the spinal cord after nerve injury. In the case of conventional neuropathic pain, sensory information from the dorsal root goes to the ipsilateral ventral horn of the injured side. In the case of mirror image pain, sensory information from the dorsal root goes to the ipsilateral ventral horn on the uninjured side. Therefore, conventional neuropathic pain and mirror image pain behaviors are represented unilaterally. However, the crossed-withdrawal reflex is represented contralaterally. As a result, sensory information from the dorsal root on the uninjured side travels across the midline of the spinal cord to reach the contralateral ventral horn of the injured side (Figure 5).

Our present study showed that electrical stimulation on the uninjured side increased metabolic activities on the injured side through the multisegments of the spinal cord. These results suggest that maybe there exist heterotopic transneuronal signaling pathways between the uninjured side and the injured side. There are evidences supporting the existence of the transneuronal signaling pathway. The anatomical study by Fitzgerald [23] reported that a neural connection exists at the spinal cord, indicating that the transneuronal signaling pathway may be mediated via commissural tracts or interneurons in the spinal cord. In a pathological condition as shown after injury, sensations on the intact side can be frequently related to the sensations on the injured side. According to an electrophysiological study by Sotgiu and Biella [20], 88% of wide-dynamic range (WDR) neurons ipsilateral to injury were activated by noxious mechanical stimulation on the intact side in anesthetized, spinalized rats. The activated responses of WDR neurons were significantly larger in nerve-injured rats than in sham or intact animals. In the case of phantom limb pain, painful muscle areas in the intact limb were related to the painful areas in the phantom limb [24]. Light stimulation on a healthy limb often leads to touch or pain in both the injured and intact limbs (synchiria) [25, 26]. Furthermore, Casale et al. [25] found that phantom limb pain can be relieved by contralateral myofascial injection of bupivacaine, a local anaesthetic, in human patients. In addition, phantom limb pain can be attenuated by transcutaneous electric nerve stimulation (TENS) applied to the contralateral healthy limb [27]. These suggest that heterotopic treatment may relieve chronic pain through the transneuronal signaling pathway.

The detailed mechanisms of the crossed-withdrawal reflex with nerve injury still remain to be determined. However, this result is in good agreement with the report which demonstrates that peripheral nerve injury, a model for neuropathic pain, is associated with a synaptic plasticity in the spinal dorsal horn [28]. The crossed-withdrawal reflex provides a useful model for neural plasticity and may contribute to the modulation of neuropathic pain.

## 5. Conclusions

We observed that the rats with unilateral nerve injury showed withdrawal responses of the injured paw to stimuli applied to the contralateral uninjured paw. We called this unusual response type “the crossed-withdrawal reflex.” We determined the “crossed-withdrawal reflex” by behavioral and electrophysiological aspects in our previous report.

In the present study, electrical stimulation of the uninjured paw of neuropathic rats produced significant increase of 2-DG uptake in contralateral ventral horn in the lumbar spinal cord. This result is in accordance with the behavioral and electrophysiological findings of the crossed-withdrawal reflex and suggests that information processing from input on the uninjured side to output on the injured side may be facilitated in neuropathic rats by neuroplastic changes through the heterotopic transneuronal signaling pathway in the spinal cord after peripheral nerve injury.
